# Low dose cadmium exposure regulates miR-381–ANO1 interaction in airway epithelial cells

**DOI:** 10.1038/s41598-023-50471-z

**Published:** 2024-01-02

**Authors:** Pooja Singh, Fu Jun Li, Kevin Dsouza, Crystal T Stephens, Huaxiu Zheng, Abhishek Kumar, Mark T. Dransfield, Veena B Antony

**Affiliations:** 1 Division of Pulmonary, Allergy and Critical Care Medicine, Department of Medicine, University of Alabama at Birmingham, Birmingham, AL USA; 2 UAB Superfund Center Advisory Board, Gainesville, FL United States

**Keywords:** Molecular biology, Pathogenesis

## Abstract

Chronic obstructive pulmonary disease (COPD) is the 3rd leading cause of death worldwide. Cigarette smoke which has approximately 2–3 µg of Cadmium (Cd) per cigarette contributes to the environmental exposure and development and severity of COPD. With the lack of a cadmium elimination mechanism in humans, the contribution of cadmium induced stress to lung epithelial cells remains unclear. Studies on cadmium responsive miRNAs suggest regulation of target genes with an emphasis on the critical role of miRNA–mRNA interaction for cellular homeostasis. Mir-381, the target miRNA in this study is negatively regulated by cadmium in airway epithelial cells. miR-381 is reported to also regulate ANO1 (Anoctamin 1) expression negatively and in this study low dose cadmium exposure to airway epithelial cells was observed to upregulate ANO1 mRNA expression via mir-381 inhibition. ANO1 which is a Ca^2+^-activated chloride channel has multiple effects on cellular functions such as proliferation, mucus hypersecretion and fibroblast differentiation in inflamed airways in chronic respiratory diseases. In vitro studies with cadmium at a high concentration range of 100–500 µM is reported to activate chloride channel, ANO1. The secretory epithelial cells are regulated by chloride channels like CFTR, ANO1 and SLC26A9. We examined “ever” smokers with COPD (n = 13) lung tissue sections compared to “never” smoker without COPD (n = 9). We found that “ever” smokers with COPD had higher ANO1 expression. Using mir-381 mimic to inhibit ANO1, we demonstrate here that ANO1 expression is significantly (p < 0.001) downregulated in COPD derived airway epithelial cells exposed to cadmium. Exposure to environmental cadmium contributes significantly to ANO1 expression.

## Introduction

COPD is associated with airflow limitation as a consequence of the combined effects of emphysema, airway wall thickening, mucus obstruction, and peribronchiolar fibrosis which presents clinically as cough, sputum production, and progressive dyspnea. The severity of these abnormalities varies between patients but a subset is particularly impacted by mucus dysfunction including hypersecretion and impaired mucociliary clearance which can lead to mucus plugging. The mechanisms underlying this mucus dysfunction are complex and incompletely understood though exposure to cigarette smoke (CS), aeroallergens, and particulate and gas pollution can contribute.

Cadmium (Cd) is a toxicant that humans are exposed to through air, food and water. Combustion releases cadmium oxide which can be easily adsorbed on the particulate matter (PM) of < 2.5 including diesel exhaust particles or CS. CS which has approximately 2–3 µg of Cd per cigarette is associated with an increased number of mucus producing cells, impaired mucus clearance and increased mucosal permeability to allergens^1^. But the mechanisms contributing to this mucosal dysfunction have many facets.

The evolutionarily conserved small non-coding RNAs (miRNAs) have been studied to determine their role in the regulation of protein translation and their contribution to disease pathophysiology^2^. miRNA or lncRNAs may be positively or negatively regulated by epigenetic regulators like EZH2. Heavy metals are not only known to induce changes in protein expression profiles but also have an effect on microRNA through epigenetic marker regulation^3^. Prediction of Cd-responsive miRNAs suggested regulation of 214 target genes^4^. ANO1 is a mechano-sensitive chloride channel, preferentially Ca^2+^ activated, encoded by the ANO1 gene located on human chromosome 11q13. A 960 amino acid protein with 10 membrane spanning segments it is expressed on the apical region of a variety of epithelial cells such as gastrointestinal, bronchial and pulmonary vessels smooth muscle cells and endothelial cells of arteries. Studies have reported regulation of ANO1 by miR-9, miR-144, miR-181a-2-3p and miR-381^5–8^. miR-9 in bronchial epithelial cells negatively regulates ANO1 expression in cystic fibrosis (CF)^9^. In a study of CF subjects, expression of miR-9 is higher which suppresses the expression of ANO1 by directly binding to the 3ʹ UTR of ANO1 mRNA. This study proposed miR-9 as a potential target for anti-CF therapy where its downregulation increases ANO1 associated chloride efflux to potentiate mucociliary clearance^5^. Also, knockout of ANO1 was shown to reduce Cl^−^ conductance and inhibit mucus secretion. Interestingly, activation of ANO1 by denufosol induced cough and failed to provide any benefit to CF patients^10^. In chronic lung diseases such as asthma and CF which have high susceptibly to environmental pollutants/allergens expression of ANO1 in airway epithelial cells is upregulated in the presence of mucus expression inducing interleukins- IL-4, IL-8 and IL-13^5,11,12^. In asthma increased ANO1 is shown to effectively modulate MUC5AC expression and mucus production. However, in COPD expression of ANO1 is not well studied. Since ANO1 supports fluid secretion and airway smooth muscle contraction, inhibition rather than activation is suggested as an appropriate treatment mechanism for inflammatory airway diseases. miR-381 was our target miRNA for this study because its association is closely related to barrier dysfunction and cell proliferation and ANO1 is most recognized for cellular functions such as cell proliferation, attachment and a controversial role in the secretion of mucus^5,13–15^. In the present work, we hypothesize that Cd negatively regulates miR-381 expression to upregulate ANO1 expression in airway epithelial cells.

## Methods

### Lung tissue sample and BALF

**Figure 1:**
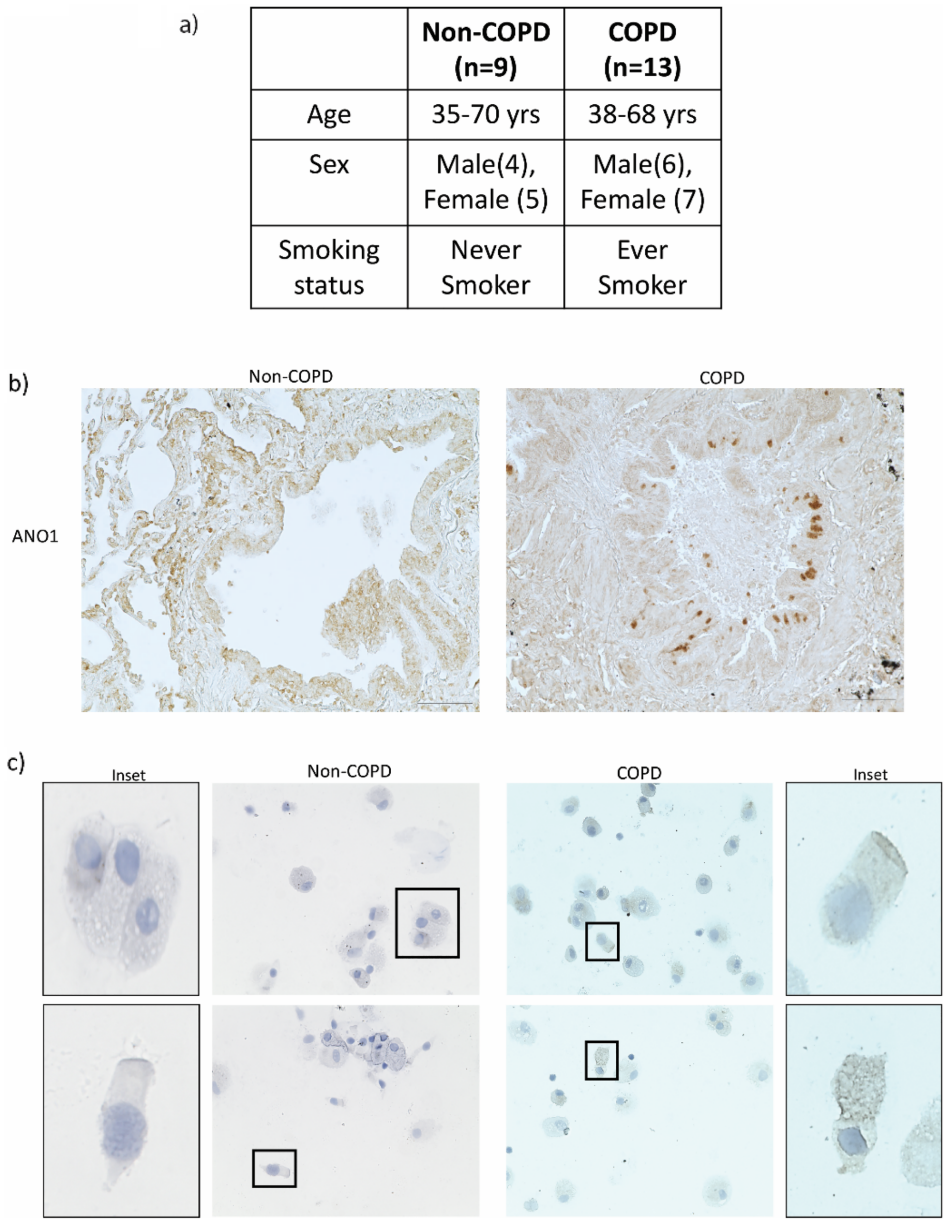
ANO1 expression in COPD. (**a**) Patient demographics with age range and smoking status. (**b**) Non-smoker healthy control (non-COPD) and smokers with COPD (COPD) lung tissue sections stained for ANO1. (**c**) Bronchoalveolar lavage fluid (BALF) derived cells stained for ANO1. Insets show ANO1 staining in airway epithelial cells. × 20 magnification.

Human lung tissue sections were obtained from the lung tissue biorepository at the University of Alabama at Birmingham as per the IRB protocols and guidelines. “Never’” smokers (n = 9) without COPD with no smoking history were selected as control subjects for the study. The test subjects were “ever” smokers (n = 13), with a past history of smoking (range of 15–25 pack years of smoking) and with COPD (Fig. 1a). Participants were categorized as non-COPD control and COPD based on the Global Initiative for Chronic Obstructive Lung Disease (GOLD)-2017 criteria, with predicted ratio of postbronchodilator forced expiratory volume in 1 s (FEV_1_) to forced vital capacity < 70% and FEV_1_ < 80%^16^. Bronchoalveolar lavage fluid (BALF) was collected from non-COPD (n = 1) and COPD (n = 1) subjects. BALF fluid collected was centrifuged to pellet the cells. Cells were suspended in PBS, counted and diluted for seeding on the glass slide using cytospin. Cells seeded on glass slide were fixed in acetone and stored in − 20 °C until further use. For ANO1 expression analysis these slides were stained with ANO1 specific antibody. Images obtained are provided in the figure sections. All experiments were performed with relevant guidelines of the protocol approved by the institutional Review Board from The University of Alabama at Birmingham, affiliated hospital. Informed consent was obtained from all subjects and/or their legal guardian(s).

### Immunohistological analysis

Tissue sections were de-paraffinized, hydrated, and after antigen retrieval, the endogenous peroxidase activity was inhibited by incubating the sections with hydrogen peroxide for 8 min. The slides were then treated with primary antibodies—rabbit polyclonal ANO1 (PA5-78778 1:500; Invitrogen, Thermo Fisher Scientific Inc., USA), for 2 h after treating sections with 3% bovine serum albumin for 20 min. Slides were washed and incubated in anti-rabbit HRP conjugated secondary antibodies at room temperature. Antigen was visualized with the InnovaRed visualization system as per the manufacturer’s protocol. All incubations were carried out at room temperature and the sections were thoroughly washed in-between incubations. Similarly, BALF derived cells seeded on glass slide were stained for ANO1 expression. Microscopy was performed using Keyence microscope (Keyence, USA) and images were taken at × 20 magnification.

### In silico analysis

List of miRNAs interacting with mRNA of ANO1 were recognized from miRNA information web-based softwares—PicTar, miRDB and TargetScan. Common miRNAs were identified, and target miRNA was selected based on interaction with ANO1 and were not affecting upstream epigenetic regulators (EZH2, SUZ12, E2F1, etc.). miR-381 was found to be interacting with all the transcripts of ANO1.

### Cell culture

Normal human airway epithelial cells (NHBECs, Lonza, Walkersville, MD) and primary human airway epithelial cells from subjects with COPD (CBECs, Lonza, Walkersville, MD) were obtained and maintained in bronchial growth medium (BEGM) from Lonza (Lonza, Walkersville, MD). For air–liquid interface (ALI) culture 24 mm-trans well inserts (Corning Costar Corporation, Cambridge, MA) were coated with collagen I, rat tail (Corning, Bedford, MA). Cells were seeded on these collagen-coated inserts at a density of 5 × 10^5^ cells/insert/well and maintained in PneumaCult Ex Plus media (Stemcell Technologies Inc, Cambridge, MA). Cells were provided fresh expansion media every day for 5–7 days in apical and basal chambers. Later media was removed from the apical chamber and differentiation media (PneumaCult-ALI Medium, Stemcell Technologies Inc, USA) was added to the basal chamber and cultures were maintained for 28 days. Every second day, the media was changed in the basal chamber and the apical region was washed with 1× PBS^17,18^. Supplementary Fig. 1 demonstrates the presence of ciliated (acetylated tubulin) airway epithelial cells in the ALI cultures. Cell cytotoxicity (MTT assay) was performed for the serially diluted CdCl_2_ solution for the epithelial cells (Supplementary Fig. 2). Cells were exposed to different concentrations (0.1, 0.5 and 1 µM) of low dose cadmium for 24 h provided in the form of CdCl_2_. Cultures were maintained for 14 days. Experiment was performed in triplicates. Membranes from the trans-well inserts were harvested and sectioned for subsequent analysis. For immunofluorescence staining inserts were fixed with 4% PFA overnight at 4 °C and rest of the sections were processed for protein and RNA isolation.

### Cell transfections

For transfection of NHBECs and CBECs cells with miR-mimic, negative control and inhibitor (Millipore Sigma) previously discussed protocol was followed^9^. Briefly, cells were grown in 6 well plates until 50–60% confluence was reached. miR-381 mimic, 5ʹ-UAUACAAGGGCAAGCUCUCUGU-3ʹ (MISSION microRNA mimic, Human, Milipore Sigma), miR-381 inhibitor (#HSTUD0174, MISSION® Synthetic microRNA Inhibitor, Human, Milipore Sigma, USA) or negative control (miR-mimic negative control) oligonucleotides were transfected into cells using Lipofectamine 2000 (Thermofisher Scientific, Inc.) according to the manufacturer’s protocol. Expression of miR-381 was estimated in transfected cells.

### Whole-mount immunofluorescent staining and analysis

Cells on trans-well insert membranes were fixed with methanol and rehydrated in decreasing concentrations of methanol to post-fix with acetone. Cells were incubated in blocking buffer (10% BSA) for 2 h at room temperature and stained with primary antibody for ANO1 (# PA5-78778 1:500; Invitrogen, Thermo Fisher Scientific, USA) in 3% BSA overnight at 4 °C. Membranes were washed in PBS and cells were counter-stained with secondary antibody. Cells were mounted with DAPI containing mounting media. Slides were examined using the Keyence BZ-X710 inverted microscope.

### RNA extraction and quantitative PCR

RNA expression analysis was performed as previously described^19^. Briefly, Total RNA was extracted using RNAeasy mini kit (Qiagen, Germantown, USA). 1 µg of RNA was reverse transcribed into cDNA with iScript Reverse Transcription SuperMix for RT-qPCR according to the manufacturer’s instructions (Bio-Rad, USA). Quantitative reverse transcription (qRT)-PCR was performed with SYBR Master Mix (Applied Biosystems, USA) on the Step one real-time PCR system (Applied Biosystems, USA). The relative expression of genes was calculated using the 2^ΔΔ^Ct method. Each experiment was performed in triplicates. miR-381 expression assay was run on the same instrument and fold change was calculated by the same method. To detect the expression of miR-381, miR *vana* micro-RNA assay kit (Invitrogen, Thermo Fisher Scientific Inc., USA) was used as per the manufacturer’s protocol. cDNA was synthesized using TaqMan microRNA reverse transcription kit and quantified using TaqMan human microRNA assay kit (Thermo Fisher Scientific Inc., USA). Relative expression was represented as fold change as normalized to endogenous reference U6 from untreated control cells. Relative expression was also used for the representation of effective transfection of miR-mimic and miR-inhibitor. Gene-specific primer pairs used were-—human ANO1 (sequence); miR-381 (F: TGGTACTTAAAGCGAGGTTGC R GGTCATGCACACACATACCAC); U6 (F: CTCGCTTCGGCAGCACA R: AACGCTTCACGAATTTGCGT).

### Western blot analysis

Sectioned membranes of ALI cultures were added to RIPA (Radioimmunoprecipitation assay) buffer (Sigma-Aldrich, USA) supplemented with protease inhibitor cocktail on ice for 1 h and centrifuged at 12,000×*g* for 10 min at 4 °C. Total protein extracted was quantified using BCA assay kit (Pierce, Thermo Fisher Sci, USA) on the spectrophotometer (BioTek, USA) at 450 nm. Equal concentrations (20 µg) of protein were used for SDS-PAGE based protein separation on 4–12% gels (Bio-Rad) and transferred on a PVDF membrane (Millipore, Sigma, USA). Membranes were blocked using Everyblot (BioRad, USA) for 20 min at room temperature, incubated with primary rabbit raised polyclonal antibody against ANO1 (1:1000; Invitrogen, Thermo Fisher Scientific, USA), mouse monoclonal antibody against β-actin (1:500; Sigma-Aldrich, USA) overnight at 4 °C. Membranes were washed with TBST for 5 min and incubated with respective HRP-conjugated anti-rabbit and anti-mouse secondary antibody (Sigma-Aldrich, USA) for 2 h at room temperature. Protein detection was done using the ECL detection system (Thermo Fisher Sci, USA) and relative quantification for band intensity was done using ImageJ software.

### Statistical analysis

All experimental data were analyzed for significance determination using GraphPad Prism 9 software. Two groups were analyzed using unpaired student t-test and for three or more groups one-way analysis of variance (ANOVA) was used with Bonferroni and Tukey post analysis; p < 0.05 was considered significant. Using Person’s correlation test correlation coefficients were derived for ANO1 and miR-381 expression.

## Results

### ANO1 expression in smokers with COPD

ANO1 expression is upregulated in chronic inflammatory diseases like asthma^17^. ANO1 expression was analyzed in lung tissue sections of smokers with COPD. ANO1 expression was observed to be upregulated (Fig. 1b) in airway epithelial cells of smokers with COPD. BALF separated cells when stained for ANO1 expression demonstrated higher ANO1 positive airway epithelial cells in smokers with COPD (Fig. 1c).

### Low dose Cd exposure induces ANO1 expression in NHBECs

**Figure 2:**
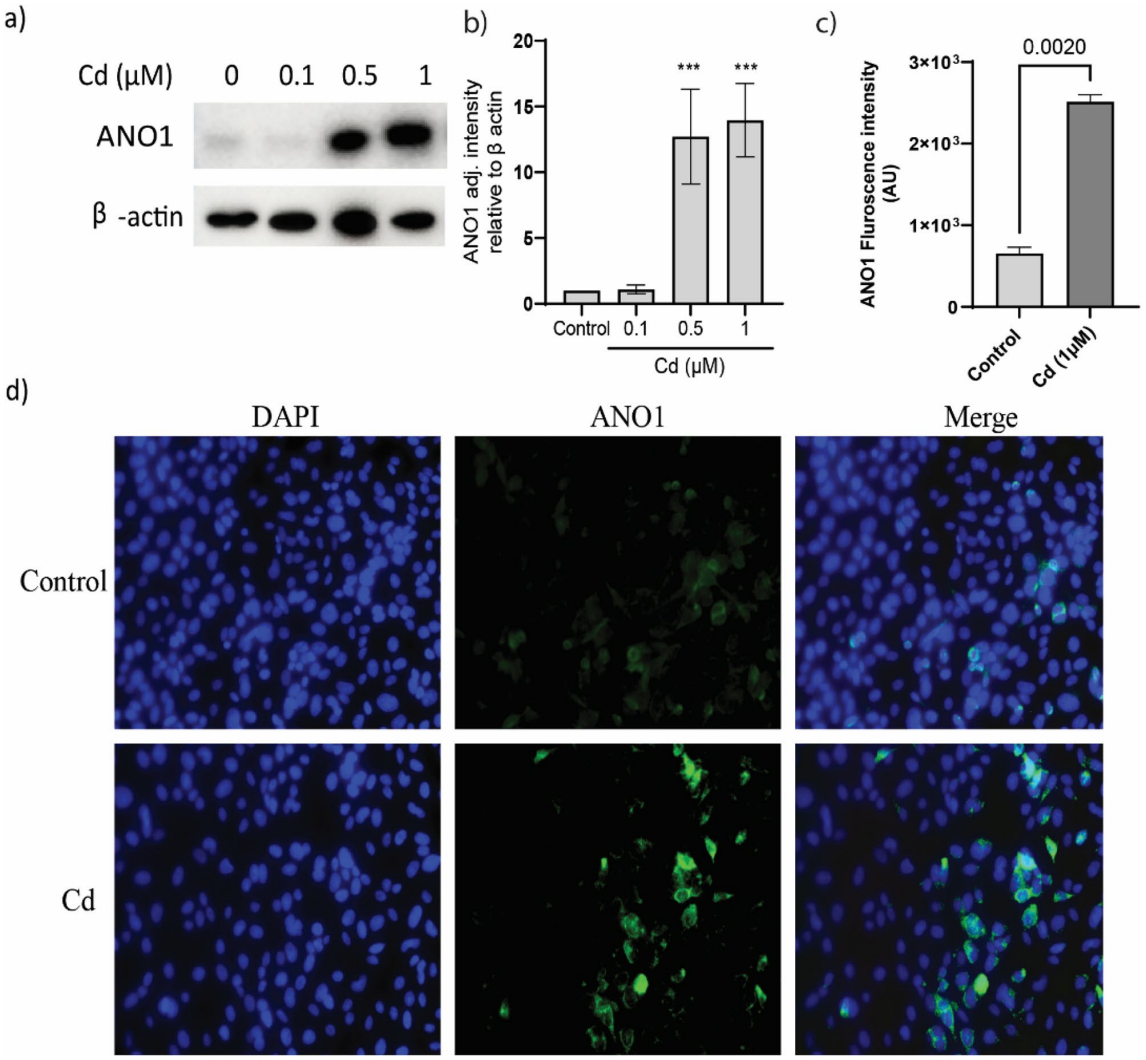
Effect of low dose of CdCl_2_ (Cd) on ANO1 expression in NHBECs. (**a**) ANO1 expression in low-dose CdCl_2_ (0.1–1 µM) exposed epithelial cells. (**b**) ANO1 intensity in western blots quantitated. Statistical significance drawn using one-way ANOVA—control vs 0.5 µM, p < 0.0001 and control vs 1 µM, p < 0.0001. ANO1 expression in Cd exposed cells represented with fluorescence microscopy at × 20 magnification (**c,d**).

ALI cultures of NHBECs were exposed to increasing concentrations—0.1, 0.5 and 1 μM of CdCl_2_ (Cd) in the basal chamber in the presence of growth medium. After 14 days of exposure ANO1 expression increased significantly (Fig. 2a,b) with increasing concentrations of Cd i.e. at 0.5 µM (adj. intensity 12.715 ± 3.609) and 1 µM (adj. intensity 13.96 ± 2.798). We further stained 1 µM CdCl_2_ exposed monolayers to demonstrate the difference in expression of ANO1 compared to control untreated cells (Fig. 2c,d). Increased number of ANO1 positive cells observed in Cd exposed cells (p = 0.002).

### miR-381 and ANO1 interaction

**Figure 3:**
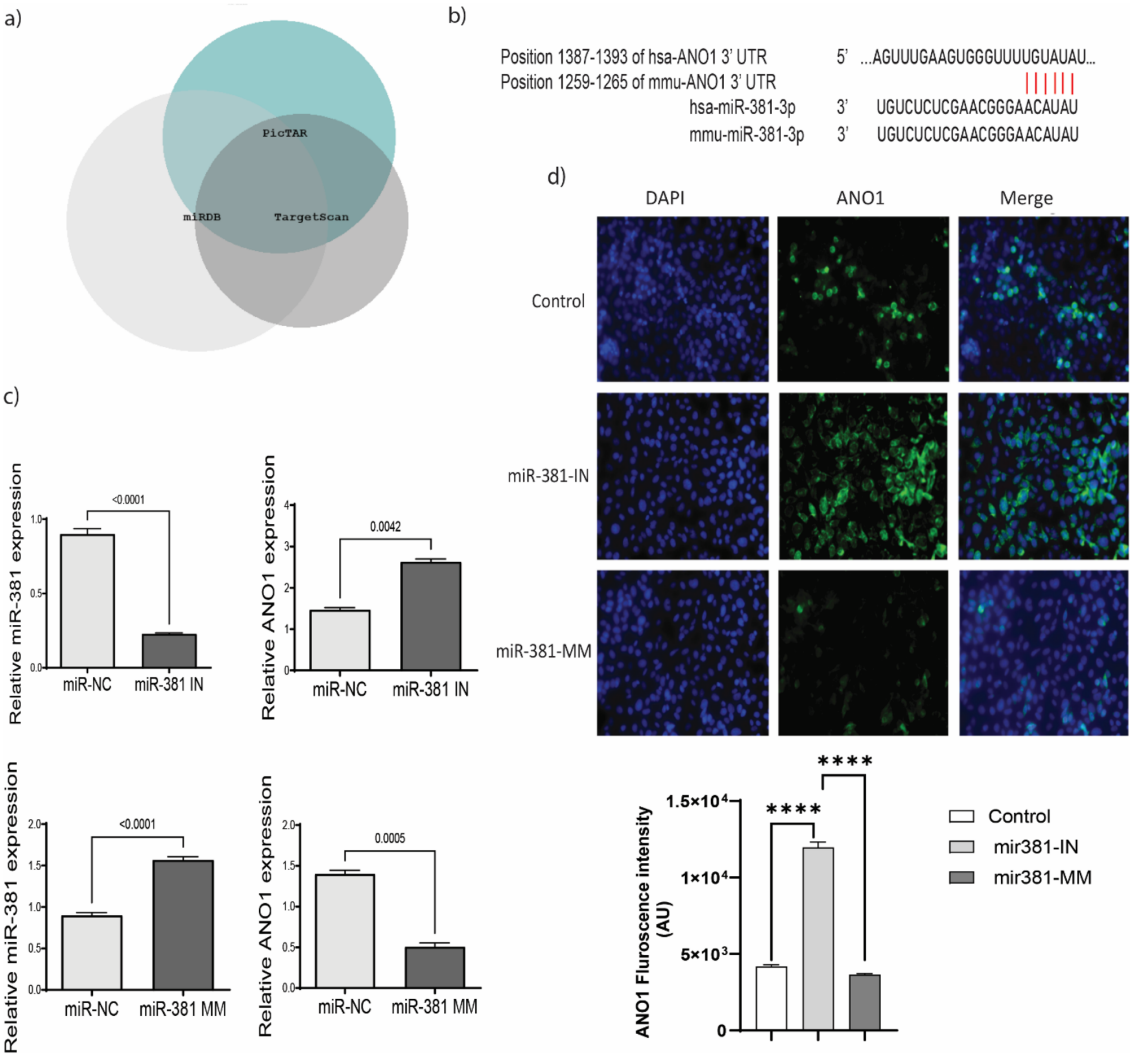
miR-381 negatively regulates ANO1 expression in Cd exposed CBECs. (**a**) miRNAs regulating ANO1 mRNA sequence were analyzed from different miRNA databases—TargetScan, miRDB and PicTAR.Venn diagram using BioVenn platform. (**b**) Putative binding sequence of miR-381 in wild type 3ʹ-UTR of ANO1. (**c**) Relative expression of miR-381 and ANO1 in presence of miR-381 inhibitor (miR-381 IN) or mimic (miR-381 MM). Expression analysis was performed by drawing comparisons against miR-381 negative control (miR-NC). (**d**) Expression of ANO1 upon miR-381 overexpression and inhibition in CBECs represented with fluorescence microscopy at × 20 magnification.

Three miRNA databases were scanned for ANO1 mRNA sequence interacting partners (Fig. 3a). Interaction analysis suggested five miRNAs common in all databases—miR-9, miR-19a, miR-19b, miR-144 and miR-381. Out of these, miR-381 sequence interacted with all the mRNA sequences of ANO1 reported in human (Fig. 3b). Using a synthetic inhibitor for miR-381 we inhibited its expression in CBECs (Fig. 3c). In these cells ANO1 mRNA expression upregulated significantly (p = 0.0042) compared to the negative control. Transfection of miR-381 mimic in CBECs decreased ANO1 expression, p = 0.0005 (Fig. 3c). Change in expression of ANO1 as also demonstrated by fluorescence microscopy in miR-381 overexpressing cells and in the presence of inhibitor for miR-381 (Fig. 3d). This observation strengthened our premise on the role of miR-381 interaction with ANO1 in Cd-exposed CBECs.

### miR-381 negatively regulates ANO1 in Cd exposed CBECs

**Figure 4:**
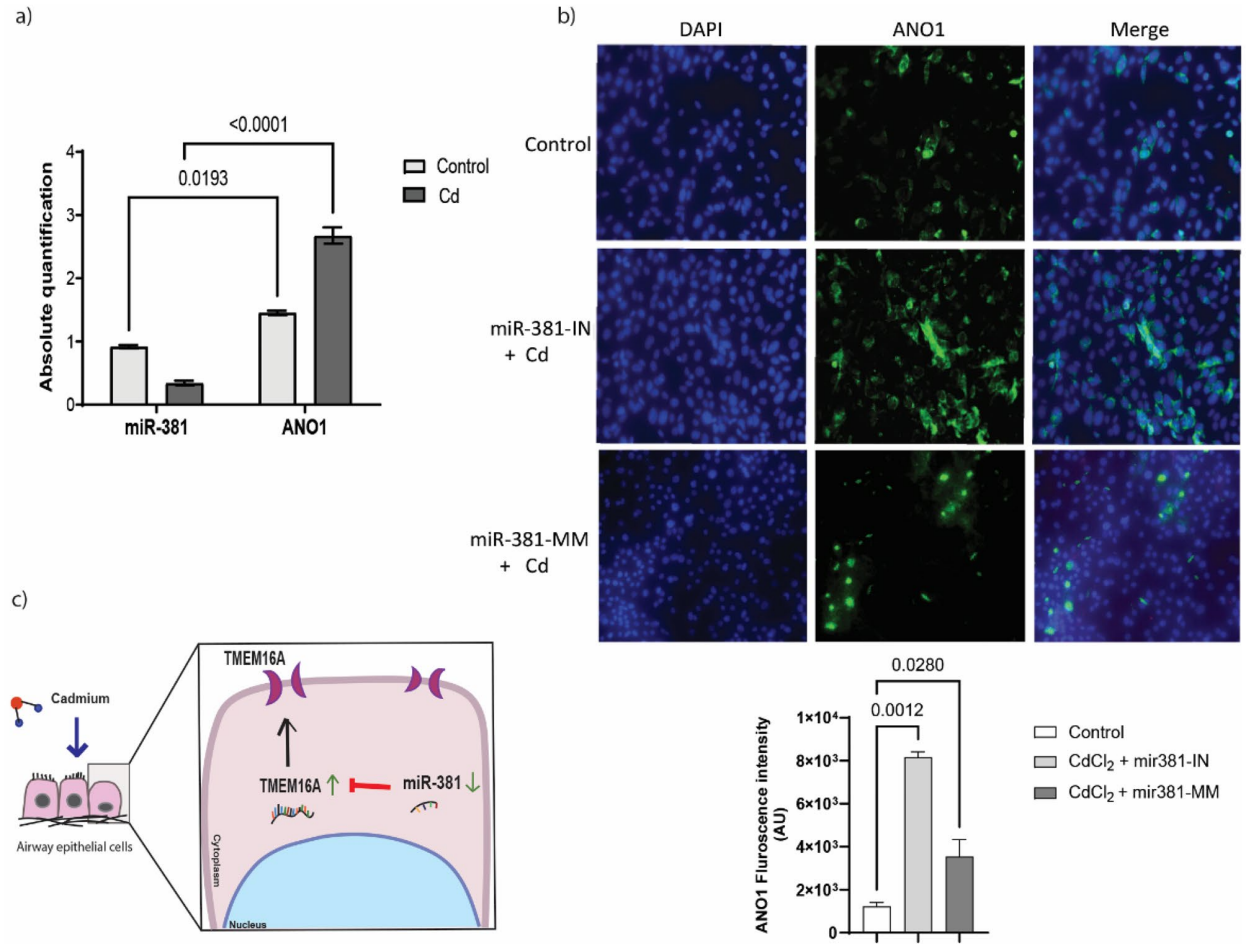
CBECs treated with CdCl_2_ (1 µM), (**a**) expression analysis for miR-381 and ANO1 using qRT-PCR. (**b**) CBECs transfected with miR-381 mimic (miR-381-MM) and control and treated with miR-inhibitor (miR-381-IN) analyzed for ANO1 expression. (**c**) Graphical abstract: Cadmium exposure to airway epithelial cells inhibits miR-381 expression which induces ANO1 expression.

miR-381 and ANO1 interaction was analyzed in the presence of Cd (1 μM) in CBECs. A negative correlation was observed between miR-381 and ANO1 mRNA with p value < 0.0001 (Fig. 4a). Also, upon miR-381 inhibition or overexpression, Cd-exposed CBECs demonstrated change in ANO1 expression. ANO1 expression was reduced in miR-381 overexpressing cells (Fig. 4b). Cd exposure in miR-381 inhibited cells demonstrated increased (p = 0.0012) ANO1 expression (Fig. 4b).

## Discussion

Environmental and occupational exposures to heavy metals is a major health concern, with acute and chronic lung toxicity. CS exposure is associated with COPD development and severity. Cd content in CS induces lung pathologies by affecting a variety of cell types in the lung. Airway epithelial cells act as the first line of cells to be exposed to exogenous particles and Cd is shown to modulate its membrane permeability. Also, pre-exposure to Cd exacerbates RSV (respiratory synical virus) infection outcomes with increased levels of IL-4 and fatty acid metabolism associated metabolites. Cd exposure also induces mucus production upon RSV infection in mice^20^. Our data presented here show that low concentrations of Cd upregulates ANO1 expression in airway epithelial cells of patients with COPD. Increase in ANO1 expression is accompanied with an increase in mucus associated proteins in asthma^12^. Thus, Cd may indirectly influence mucus secretion in airway epithelial cells by regulating the expression of ANO1. Another important observation in the present study was the decrease in miR-381 level and the overexpression of miR-381 sequence resulted in inhibition of Cd-induced ANO1. Heavy metals are not only known to induce changes in protein expression profiles but also influence microRNA^3^. This suggests miRNA mediated regulation of ANO1 in chronic inflammatory conditions such as asthma, COPD and CF where inhibition of ANO1 rather than activation is being preferred. Also, Air liquid interface cultures are an important tool for studying differentiated airway epithelial cells. In a study, COPD patient derived sinonasal brushing samples were compared with ALI-cultured nasal epithelial cells and observed 96% similar genetic expression profile^21^. ANO1 is expressed on the apical region of epithelial cells and therefore ALI cultures served as better model for this study.

The chloride channels can be regulated or are sensitive to thermal stimuli, cell volume, Cd–metal ions and calmodulin^15^. Cd is an activator, with low sensitivity, of ANO1 at a concentration range of 100–500 µM in in vitro^15,22^. At concentrations higher than 500 µM (~ 1000 µM) experimental Cd bridge is shown to deactivate ANO1 channel by hiding the Ca^2+^ binding sites^23^. In vivo observations with Cd-exposed airway epithelial cells display airway remodeling which is also a characteristic of inflammatory diseases like asthma and COPD due to prolonged and uncontrolled inflammation^24–27^. Our results suggest a close association between ANO1 expression and miR-381 in the airways of COPD patients. Compared to control subjects with no smoking history, smokers with COPD have an increase in ANO1 expression in the airways. Cd-exposure is not only confined to active smokers but its lack of elimination from the body (~ 26+ years) puts past smokers and even passive smokers at risk of developing Cd-induced cellular defects. It is therefore an important observation as patients with COPD display increased mucus production and accumulation and a regulatory mechanism is required to overcome this defect. The stages of COPD were not considered in the present work as the scope was to understand the regulatory mechanism for ANO1 expression in the airway epithelial cells exposed to a low dose of Cd. One limitation of our study is the lack of information on subject corticosteroid usage, since that might alter inflammatory changes.

ANO1 can be regulated through mRNA–miRNA interaction. miR-381 was the target miRNA tested for the correlation in this study and our results suggest an inverse correlation between miR-381 and ANO1 expression. Inhibition or upregulation of miRNAs using synthetic inhibitors or mimic has been shown to be an effective approach rather than targeting upstream pathways to achieve endogenous regulation of miRNAs. In this study overexpression of miR-381 using a mir-381 mimic had a negative effect on ANO1 expression in Cd-exposed cells. This suggests that targeting miR-381 can serve as a regulatory mechanism for ANO1 (Fig. 4c).

TGF-β signaling which has been extensively studied and reported to highly expressed and promote airway remodeling in asthma is also suppressed by an increased expression of miR-381. TGF-β promotes goblet cell transformation resulting in increased mucus production and secretion^28^. Knockdown of miR-381 increases epithelial cell proliferation with increased Ki-67 stained cells and intensity^13^. In gastric cancer induction of miR-381 expression suppresses differentiation of epithelial cells and reduces cell proliferation^29^. Our observations from experiments involving low-dose Cd-exposure of epithelial cells suggested a similar understanding that ANO1 is a direct target for miR-381 which is downregulated upon low-dose Cd-exposure (Fig. 4c). Thus, CS-induced Cd-toxicity may alter cellular homeostasis mechanisms at very low concentrations. Thus Cd-exposure in a person with an existing pulmonary condition can have an additive or adverse effect with increased susceptibility towards infections and environmental allergens and that miRNAs may act as potential therapeutic targets to be explored further in Cd-exposure and subsequent lung injury.

### Supplementary Information

Supplementary Figures.

## Data Availability

The datasets generated during the current study are available from the corresponding author on reasonable request.

## Abbreviations

CACCs: Calcium activated chloride channels
COPD: Chronic obstructive pulmonary disease
ANO1: Anoctamin 1
miR: Micro RNA
