# Supplementary material for: Low dose cadmium exposure regulates miR-381–ANO1 interaction in airway epithelial cells
Source: Sci Rep. 2024 Jan 2;14:246. doi: 10.1038/s41598-023-50471-z (PMC10762153; doi:10.1038/s41598-023-50471-z)
Supplement: Supplementary file 1 — Supplementary Figures. [file 41598_2023_50471_MOESM1_ESM.pdf]

Supplementary Figures

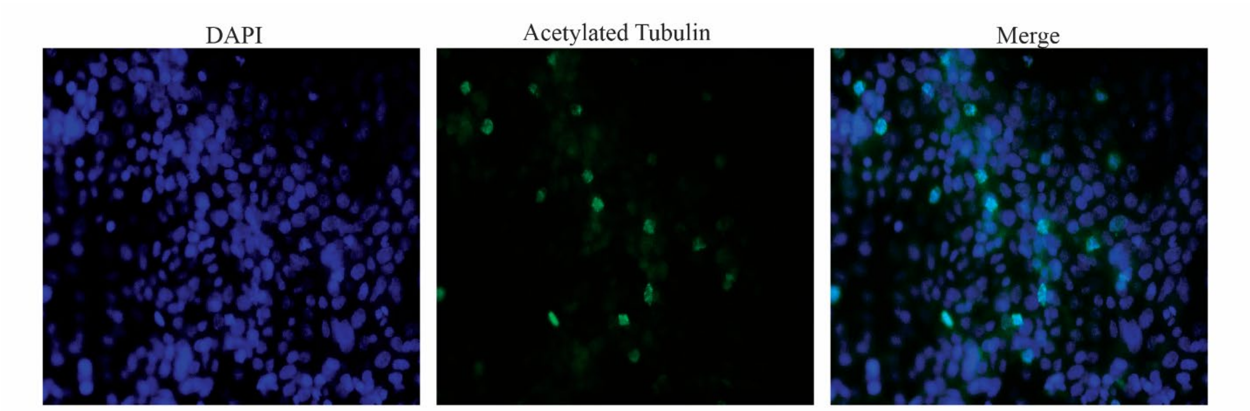

Supplementary Figure 1: Airway epithelial cells grown in air liquid interface conditions. The images demonstrate acetylated tubulin marker for cilia and its presence across the cultured cells as observed under 20x magnification.

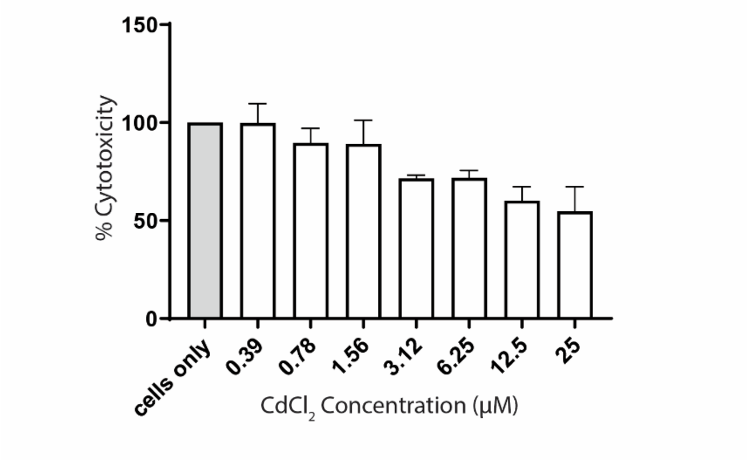

Supplementary Figure 2: Cadmium cytotoxicity performed for CBECs at different serial dilutions. O.D. was measured at 540 nm.

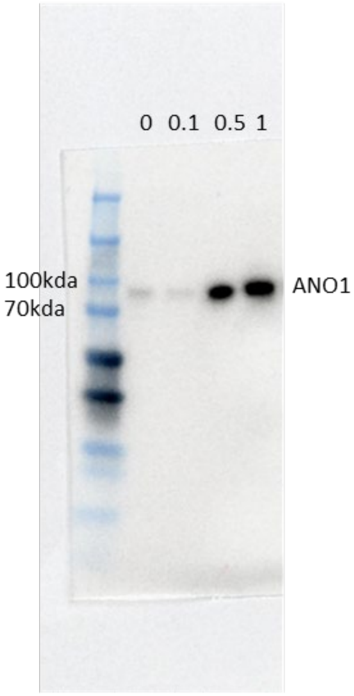

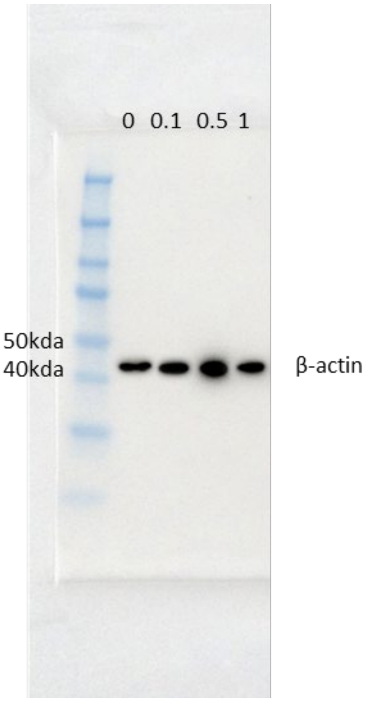

Supplementary Figure 3: Uncropped western blot images for ANO1 (1:1000; Invitrogen, Thermo Fisher Scientific, USA) and β-actin (1:500; Sigma-Aldrich, USA) shown in figure 2a. ANO1 expression in low-dose $CdCl_2$ (0.1-1μM) exposed epithelial cells.
